# The effect of sexual health education on sexual activity, sexual quality of life, and sexual violence in pregnancy: a prospective randomized controlled trial

**DOI:** 10.1186/s12884-021-03803-8

**Published:** 2021-04-26

**Authors:** Shiva Alizadeh, Hedyeh Riazi, Hamid Alavi Majd, Giti Ozgoli

**Affiliations:** 1 Mother and Child Welfare Research Center, Hormozgan University of Medical Sciences, Bandar Abbas, Iran; 2 Department of Midwifery and Reproductive Health, School of Nursing and Midwifery, Shahid Beheshti University of Medical Sciences, Tehran, Iran; 3 Department of Biostatistics, School of Allied Medical Sciences, Shahid Beheshti University of Medical Sciences, Tehran, Iran; 4 Department of Midwifery and Reproductive Health, School of Nursing and Midwifery, Shahid Beheshti University of Medical Sciences, Tehran, Iran

**Keywords:** Sexual violence, Quality of life, Sexual activity, Pregnancy, Sexual health, Training

## Abstract

**Background:**

Some women avoid sexual intercourse during pregnancy due to the physiological changes they undergo during this period as well as their fear of causing harm to the fetus and to themselves, which can lead to problems in sexual health. The aim of the present study was to investigate the effects of a sexual health education package on the dimensions of sexual health in pregnant women.

**Methods:**

This randomized, longitudinal, clinical trial was carried out in 2018–2019 on 154 pregnant women in early to late pregnancy who presented to comprehensive health centers in Rasht, Iran, and were divided into three groups: Group A or the training group (50 participants), Group B or the self-training group (53 participants), and Group C or the control group (51 participants). The study tools included the Pregnancy Sexual Response Inventory (PSRI), the Sexual Quality of Life-Female (SQOL-F) and the Sexual Violence Questionnaire. The dimensions of sexual health were examined before beginning each intervention in each trimester of pregnancy and then at the end of pregnancy using these questionnaires. The collected data were analyzed using statistical tests, namely the Chi-square test, one-way ANOVA, Cochrane’s test, and the repeated measures ANOVA at a significance level of *P* < 0.05.

**Results:**

There was no statistically significant difference in the mean total scores of SQOL-F and PSRI in the three groups at baseline. As for the intergroup results, there was a statistically significant difference in the mean score of SQOL-F and PSRI at the end of pregnancy. The mean scores of PSRI and SQOL-F in the training group (Group A) increased from the beginning to the end of pregnancy compared to the control and self-training groups. As for the intergroup comparisons, there was no statistically significant difference in the mean total scores of sexual violence among the pregnant women in the different groups in the third trimester of pregnancy and at the end of the third trimester. Although sexual violence was not statistically significant, the number of sexually-violated women in the training group decreased during the training period compared to the self-training and control groups.

**Conclusion:**

The results obtained in the intervention group compared to the control group revealed the effectiveness of the sexual health education package in terms of improvement in the dimensions of sexual health. According to the results, in order to maintain and promote the sexual health of pregnant women, health care providers are recommended to offer sexual health training during pregnancy along with other health care services.

**Trial registration:**

IRCT20190427043398N1; the trial was registered on June 2, 2019. (retrospective registration).

## Background

Pregnancy is more than merely a biological transformation in a woman’s body; rather, the conception of a fetus in a woman’s uterus is the most intimate, emotional, and personal period in her life during which her body undergoes major physiological, anatomical, and even behavioral changes [1]. The physiological impact of pregnancy also brings about changes in expectant mothers’ sexual and marital relationships [2–4] and sexual behaviors [3, 5]. During pregnancy, the sexual desire and activity of pregnant women and their husbands may either increase, decrease or remain unchanged. As a result, pregnancy can either improve the marital relationship or cause it to break up [6]. Often, as pregnancy progresses, the woman’s pain increases and her sense of attractiveness, frequency and duration of intercourse, sexual desire, ability to reach orgasm, and sexual satisfaction decrease [7]. The reduction in the frequency of intercourse is often more significant in the third trimester of pregnancy [8]. The decline in sexual activity and the resultant failure to meet the husband’s sexual needs may lead some men to engage in other sexual behaviors during their spouse’s pregnancy, including extramarital sex [9–11]. A pregnant woman’s refusal to have sexual intercourse with her husband can lead to domestic violence or make an existing abusive relationship worse [9, 10, 12]. Sexual violence includes non-consensual or unwanted sexual acts using physical force or subjugation to humiliation or violence during sexual intercourse [13]. The global prevalence of violence during pregnancy is reported as 3 to 31% [3].

Sexual health during pregnancy can be affected by the mental and emotional damage caused by violence, frigidity, female depression [1], husband’s sexual unfulfillment [9, 10, 12], and sexual disorders. These often-common pregnancy-related challenges can be exacerbated by a variety of factors, including misconceptions or lack of accurate information [14]. Sexual health, which can be defined as mutual satisfaction, pleasure and meeting the sexual and emotional needs of both parties, can result in an overall improvement of the couple’s relationship. Furthermore, the decline in a couple’s sexual health during pregnancy can reduce the couple’s quality of sexual life [15, 16].

Proper sexual health training can be a strategic solution for enhancing the sexual quality of life during pregnancy. It can also serve as an effective intervention for preventing pregnancy-related sexual problems and promoting the sexual health of pregnant women and their partner [17]. Sexual health training designed to educate couples about their sexual health and ideal sexual functions can improve marital relationships and prevent the breakdown of families [18].

Any training provided about sexual health during pregnancy should be based on credible and accredited resources and be frequently repeated, such as is the case for breastfeeding and personal hygiene training, which are repeated at different months during all the pregnancy care visits made to healthcare centers.

Considering the importance of sexual health, which is part of the fourth goal of the United Nations’ Sustainable Development Goals (SDG) by 2030, the present study was conducted to investigate the effect of a sexual health education package on the dimensions of sexual health in pregnant women. During this research, as part of routine prenatal care, the researchers trained pregnant women using the Sexual Health Education Package (SHEP) formerly validated and found to be reliable. SHEP was shared with the examined pregnant women in group training and self-training groups.

The effect of the intervention on the various dimensions of sexual health was examined by different educational methods.

The research hypotheses included:

1. Group training leads to a reduction in sexual violence, improvement in sexual activities, and the enhancement of the sexual quality of life.
2. Self-training leads to a reduction in sexual violence, improvement in sexual activities, and the enhancement of the sexual quality of life.
3. Both the group training and self-training method show similar results concerning their impact on reduction in violence, improving sexual activity and enhancing the sexual quality of life.

## Methods

The present randomized and clinical trial was conducted on pregnant women presenting to the comprehensive health centers of Rasht (northern Iran) from September 2018 to May 2019. The aim of the study was to investigate the effect of a sexual health education package on the dimensions of sexual health in pregnant women.

**Primary outcomes:** Sexual activity and response during pregnancy.

**Secondary outcomes:** The rate of sexual violence in pregnancy and the sexual quality of life during pregnancy.

### Sample size

The sample size was calculated based on α = 0.05 and β = 0.20 and effect size = 0.6. The minimum required sample for each group was 45; to take account of a 40% potential drop based on studies conducted on the rate of sexual violence during pregnancy in Iran, in which the participant withdrawal rates were 31% [19] and 51.6% [20], the appropriate number of samples per group in this study was estimated as 75 (making for a total of 225 participants).

$$ n\ge 2\frac{{\left({z}_{\alpha }+{z}_{\beta}\right)}^2{\sigma}^2}{{\left({\mu}_1-{\mu}_2\right)}^2} $$

$$ \alpha =0.05\Rightarrow {z}_{\alpha }=1.96\kern0.5em \beta =0.20\Rightarrow {z}_{\beta }=0.84\kern0.5em effect\kern0.34em size=\frac{\mu_1-{\mu}_2}{\sigma }=0.6 $$

$$ n=2{\left(1.96+0.84\right)}^2{\left(\frac{1}{0.60}\right)}^2=45 $$

### Inclusion and exclusion criteria

The inclusion criteria were being a woman aged 15–45 years, having a gestational age less than 14 weeks, signing a written consent form to participate in the study, having no history of medical diseases such as diabetes and hypertension, no history of medicine consumption interfering with sexual function, no drug addiction, having a normal low-risk pregnancy (without complications at birth or the need to take certain medications based on the medical and maternal history), not having experienced acute stress in recent years, such as the death of a child, and not having any acute illnesses or marital problems or unexpected problems potentially arising during the study. The exclusion criteria included a reluctance to continue participation in the study or a high-risk pregnancy (such as spotting, premature labor, etc.).

### Group allocation and intervention

After obtaining the necessary permission, the selected comprehensive health service centers of Rasht were visited and 225 participants were randomly selected (75 per group) from those in their first trimester of pregnancy (up to 14 weeks of gestation). All the eligible candidates entered the study after they were fully briefed on the purpose of the study and signed written consent forms. For this study, random cluster sampling was performed in Rasht, Iran, and the city was divided into five regions (east, west, north, south, and center). Then, three areas of the city, including east, west and center, were randomly selected. In the next step, the participants were randomly assigned to the different groups. Using a random number function, first, 225 random numbers were assigned to three groups of 75, labelled with letters A, B and C, and then, by sorting these random numbers, the three letters A, B, and C were completely arranged in a random way. According to this random order, the samples from each of the comprehensive health centers were assigned to the three study groups (Excel file is attached as pdf).

In order to prevent sampling bias and intervention bias, the intervention schedule was adjusted in a way that contact between the samples was kept to a minimum. Efforts were also made to select the health centers in each cluster (region) from the same urban area with comparable socioeconomic and cultural backgrounds.

The Sexual Health Education Package (SHEP) designed based on the National Institute for Health and Clinical Excellence (NICE) guidelines had two parts –one for healthcare providers and one for pregnant women. The educational materials used during pregnancy (one training session for each trimester of pregnancy) included the most important dimensions of sexual health. The content of the package included notes about the components of the reproductive system of men and women, physiological changes during pregnancy, emotional and psychological changes during pregnancy, stages of the sexual response cycle and physical and psychological differences between men and women in the sexual response cycle, sexual activity and its changes in pregnancy, the factors affecting sexual activity in pregnancy, personal and sexual hygiene tips in pregnancy by trimester, pre-sex preparation and sexual intimacy, marital satisfaction, different sex positions suitable for each trimester of pregnancy, sexual restrictions during pregnancy, the right beliefs and attitudes about sexual function and correcting false beliefs and attitudes, domestic violence during pregnancy, sexual changes after delivery and the best time to restart sexual activity after delivery. The researcher presented the educational material to the participants in the self-training group using a pamphlet compiled in a simple language.

#### Group a (the first intervention group)

Group A received group training using SHEP in three 90-min sessions (one during each trimester of pregnancy). The classes were divided into small groups of 5 to 12 participants. The first session (before 14 weeks of gestation), the second session (up to 28 weeks of gestation) and the third session (up to 32 weeks of gestation) were all at least 4 weeks apart. Initially, after briefing them on the study objectives and obtaining their written consent, the participants were given some assessment questions through a demographic form and some questionnaires. Then, the classes were held based on SHEP in the form of a lecture with questions and answers to actively engage the participants. At the end of each class, the women were given a booklet to review and share with their spouses. In the last month of pregnancy (36–40 weeks of gestation), the questionnaires were given to the women again to check and assess their sexual health.

#### Group B (the second intervention group)

Group B entailed self-training using SHEP at each trimester (first trimester before 14 weeks, second trimester before 28 weeks, and third trimester up to 32 weeks of gestation). Also, the participants in Group B were given questionnaires to fill out during each trimester (with the occasions being set at least 4 weeks apart) and also at the end of their pregnancy (36–40 weeks of gestation).

#### Group C (the control group)

Group C received routine care, with no sex education material. The routine care included maternity care according to the National Guidelines for Providing Midwifery and Childbirth Services by the Ministry of Health of Iran, guidelines for pregnancy care and the guidelines presented by the World Health Organization, such as those on vaccination, blood pressure control, weight measurement, and nutrition counseling. In this group, the mothers were only given the questionnaires to complete each trimester and at the end of their pregnancy (36–40 weeks of gestation), with the four measurement occasions being at least 4 weeks apart.

**Fig. 1:**
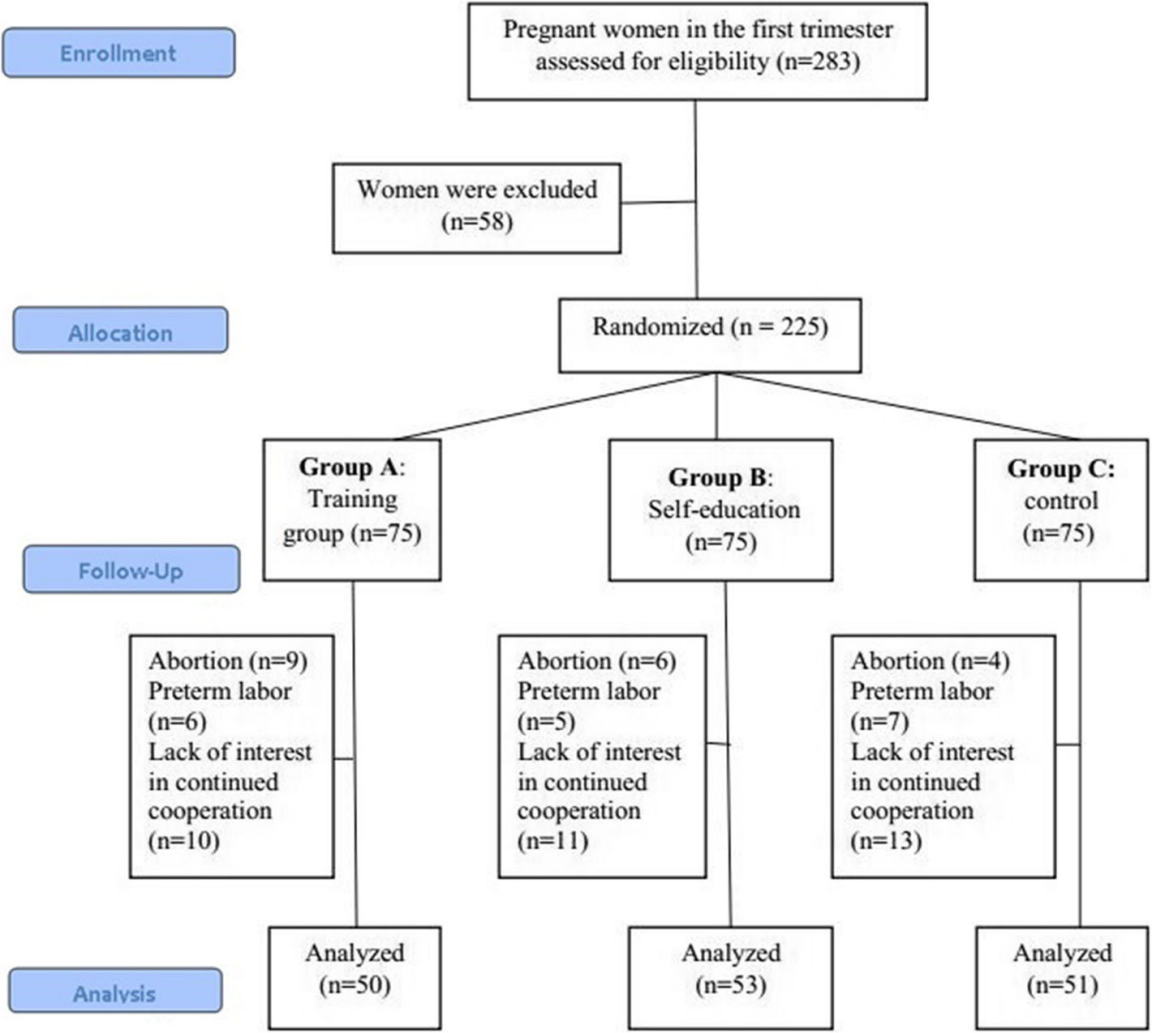
The flow diagram of the progress through the phases of the clinical trial

At the beginning of the study, 75 participants were randomly assigned to each group. Some participants withdrew from the study due to miscarriage, preterm delivery, relocation, and reluctance to continue participation (Fig. 1).

### Questionnaires

The questionnaires used in this study included a demographic form and the Pregnancy Sexual Response Inventory (PSRI), the Sexual Quality of Life-Female (SQOL-F), and the sexual violence questionnaire. The PSRI has been designed by Ruge et al. [21] and includes 26 items on sexual activity and responses in ten domains, including the frequency of sexual intercourse, sexual desire, sexual arousal, orgasm, female sexual satisfaction, dyspareunia, sexual initiation, female sexual problems, male sexual satisfaction, and male sexual problems before and during pregnancy. The total score of the PSRI is calculated out of one hundred [21], and a score below 25 indicates a very bad sexual relationship and activity, a score of 25–50 indicates poor relationship and sexual activity, 50–75 good sexual relationship and activity, and 75–100 excellent sexual relationship and activity [22]. After obtaining the written permission of the designer of the original questionnaire, it was localized for use in Iran and its validity was determined through the face validity, content validity, construct validity and reliability assessment methods. To determine the reliability and internal consistency of the questionnaire, Cronbach’s alpha coefficient and test-retest repeatability were measured [23]. The questionnaire reliability was reported as 0.891 after 30 eligible subjects filled out the localized version. Investigating stability and test-retest repeatability require an interval of 2 weeks to 1 month between the tests [24]. In this study, the questionnaire was assessed 2 weeks apart in the 30 subjects in this stage, and the scores obtained in the test and retest were compared using the Intraclass Correlation Coefficient (ICC), which was obtained as 0.97.

#### Sexual quality of life-female (SQOL-F) questionnaire

This questionnaire is the Persian version of the Sexual Quality of Life-Female (SQOL-F) questionnaire. The questionnaire was first designed in 1998 and reviewed and validated in 2005 by Symonds et al. [25]. The SQOL-F questionnaire is an 18-item scale rated on a 6-point Likert-type scale. Items 1, 5, 9, 13 and 18 in it should be graded in reverse. The minimum score obtained in this tool is 18 and the maximum 108. This questionnaire has four domains, including psychosexual feelings (including seven items: 2, 3, 7, 8, 10, 16, 17), sexual and relationship satisfaction (including five items: 1, 5, 9, 13, 18), self-worthlessness (includes three items: 4, 6, 15) and sexual repression (including three items: 11, 12, 14). The overall score of the sexual quality of life is more indicative of the desired quality of sexual life. According to the range of responses, a score up to 36 is classified as poor, a score of 37–72 as average, and 73–108 as good. Masoumi et al. translated and validated this questionnaire and reported its reliability as 0.777 using Cronbach’s alpha [15].

#### The sexual violence questionnaire

This questionnaire is part of the domestic violence questionnaire and has five items. It is scored on the basis of a five-point Likert scale (never, once, twice, three to five times, and more than five times). A woman is considered to have been subject to sexual violence if she gives at least one positive answer to the items in this questionnaire. This questionnaire was validated by Ahmadi et al. (2015) with an internal reliability coefficient of 0.99 for domestic violence [26]. The reliability of the questionnaire was assessed in this study by distributing it among 30 eligible pregnant women, and a Cronbach alpha of 0.765 was thus obtained.

### Data analysis

Data were analyzed in SPSS software version 23. In the descriptive statistics section, descriptive indicators such as mean and standard deviation were reported. The normal distribution of the data was checked using the Kolmogorov-Smirnov test. The Chi-square test and one-way ANOVA, followed by Tukey’s post-hoc test, were used to compare the three groups, and the intragroup comparisons between the four measurement occasions (i.e., before the intervention and in the first, second and third trimesters of pregnancy) were carried out using Cochrane’s test and the repeated-measures ANOVA. *P*-values less than 0.05 were considered statistically significant. The analysis was performed on the per-protocol population.

### Ethical considerations

This study was approved by the ethics committee of Shahid Beheshti University of Medical Sciences under the code IR.SBMU.PHNM.1395.495. The project was registered at the Iranian Registry of Clinical Trials under the code IRCT20190427043398N1. After providing the necessary explanations and information about the study, permission was obtained from the main designers of the questionnaires to use their tools in the study, and written consent was also obtained from the participants for taking part in the research.

## Results

A total of 154 pregnant women divided into three groups participated in this study. The mean age of the subjects was 28.30 ± 5.49 in the group training group, 28.64 ± 4.39 in the self-training group and 29.37 ± 4.92 in the control group. The level of education of most of the participants and of their spouses was high school diploma and below in all the three groups. Most of the women surveyed in all the groups were unemployed and the majority of their spouses were self-employed. The difference between the three groups was not statistically significant in terms of the demographic, social, and midwifery characteristics based on the one-way ANOVA, Chi-square test, and the Kruskal-Wallis test. Therefore, the random allocation used to divide the subjects among the three groups can be said to have homogenized the groups in terms of the demographic characteristics. To sum up, the participants had similar conditions, the method of selection used in the study was appropriate and the results are therefore reliable [27, 28] (Table 1).

**Table 1:** The sociodemographic characteristics of the participants in the three groups: A, B, C

Variable	Group A	Group B	Group C	***P***-Value
	Mean ± SD or N (%)	Mean ± SD or N (%)	Mean ± SD or N (%)	
Age				
Pregnant woman	28.30 ± 5.49	28.64 ± 4.39	29.37 ± 4.92	0.319^a^
Husband	33.54 ± 5.31	32.64 ± 4.34	33.45 ± 4.99	0.59^a^
Pregnancy number	1.82 ± 1.00	1.79 ± 0.74	1.61 ± 0.69	0.506^a^
Duration of marriage	7.04 ± 4.78	6.02 ± 4.12	6.10 ± 3.88	0.08^a^
Pregnant Woman’s Education				
High school diploma or less	37 (74)	35 (66)	27 (52.9)	0.706^b^
Associate or bachelor’s degree	11 (22)	15 (28.3)	14 (27.5)	
Master’s degree or PhD	2 (4)	3 (5.7)	10 (19.6)	
Husband’s Education				
High school diploma or less	35 (70)	39 (73.6)	26 (51)	0.904^b^
Associate or bachelor’s degree	13 (26)	12 (22.6)	20 (39.2)	
Master’s degree or PhD	2 (4)	2 (3.8)	5 (9.8)	
Pregnant Woman’s Occupation				
Unemployed	45 (90)	48 (90.6)	35 (68.6)	0./3^c^
Employed	5 (10)	5 (9.4)	16 (31.4)	
Husband’s Occupation				
Employed	5 (10)	8 (15.1)	9 (17.6)	0.438^c^
Self-Employed	26 (52)	30 (56.6)	31 (60.8)	
Other	19 (38)	15 (28.3)	11 (21.6)	

^a^The one-way ANOVA

^b^Kruskal-Wallis test

^c^The chi-square test

The intragroup comparison carried out using the repeated-measures ANOVA showed significant differences in the mean total score of PSRI between the three groups throughout the study (*P* < 0.001). Over time, the mean score of PSRI increased in group A; meanwhile, in groups B and C, the mean score of PSRI decreased. In other words, the group training program had a positive effect on sexual responses in pregnant women during the intervention.

The intergroup comparison using the one-way ANOVA showed no significant differences between the three groups in the mean total score of PSRI before pregnancy, in the first trimester (before the intervention) and in the second trimester (*P* > 0.05). Moreover, there was a notable difference among the three groups in the mean total score of PSRI during the third trimester and at the end of the third trimester (*P* < 0.001). According to Tukey’s post-hoc test, there was a significant difference in the mean total scores of PSRI between groups A and C and also between groups A and B (*P* < 0.001), but there was no significant difference in the mean total scores of PSRI between groups B and C (*P* > 0.05) (Table 2). The descriptive statistics using the repeated-measures ANOVA for the mean score of PSRI during the study period in the three groups showed that the effect of time (*P* < 0.001), groups (*P* <0.001) and interaction between time and group (*P* <0.001) were statistically significant. Significance of the interaction effect shows that the trend of (sexual) response significantly differed among the study groups over time.

**Table 2:** The trend of the total PSRI scores in the different groups (A = 50, B = 53, C = 51) during pregnancy

Time	Before pregnancy	1st trimester	2nd trimester	3rd trimester	End of pregnancy	Total	***P***-value^a^
Groups	Mean ± SD	Mean ± SD	Mean ± SD	Mean ± SD	Mean ± SD	Mean ± SD	
**Group A**	71.47 ± 16.84	45.12 ± 16.26	53.76 ± 17.83	57.68 ± 18.79	57.04 ± 16.73	57.01 ± 19.17	*P* < 0.001
**Group B**	71.55 ± 14.03	50.54 ± 18.62	51.63 ± 13.89	45.77 ± 14.59	36.92 ± 14.16	51.29 ± 18.87	*P* < 0.001
**Group C**	73.48 ± 13.56	45.25 ± 19.00	49.18 ± 16.91	42.59 ± 17.57	35.37 ± 14.80	49.17 ± 20.88	*P* < 0.001
**Total**	72.17 ± 14.79	47.01 ± 18.08	51.53 ± 16.28	48.60 ± 18.13	42.98 ± 18.07	52.49 ± 18.01	
**Effect Size**	0.13	0.30	0.28	0.83	1.20	0.43	
***P*-Value**^b^	*P* = 0.75	*P* = 0.24	*P* = 0.36	*P* < 0.001¥	*P* < 0.001¥		

^a^The repeated measures ANOVA

^**b**^The one-way ANOVA

¥: According to Tukey’s post-hoc test: There was a significant difference between the mean total scores of PSRI and Group A-C and Group A-B (*P* < 0.001) but there was no significant difference between the mean total scores of PSRI and Group B-C (*P* > 0.05)

The intergroup comparisons using the one-way ANOVA also showed no significant differences among the three groups in the mean total score of SQOL-F in the first trimester (before the intervention) and second trimester (*P* > 0.05). According to Tukey’s post-hoc test, there was a significant difference in the mean total scores of SQOL-F between groups A and C and between groups A and B (*P* < 0.05), but there was no significant difference in the mean total scores of SQOL-F between groups B and C (*P* > 0.05). The intragroup comparisons using the repeated-measures ANOVA showed a significant change in the mean total score of SQOL-F throughout the study in groups B and C (*P* < 0.001) (Table 3). The descriptive statistics using the repeated-measures ANOVA for the mean score of SQOL-F during the study period in the three groups showed that the effect of time (*P* <0.001), groups (*P* = 0.032) and interaction between time and group (*P* <0.001) were statistically significant. Significance of the interaction effect shows that the trend of sexual quality of life significantly differed among the study groups over time.

**Table 3:** The trend of total sexual quality of life scores in the different groups (A = 50, B = 53, C = 51) during pregnancy

Time	1st trimester	2nd trimester	3rd trimester	End of pregnancy	Total	***P***-Value^a^
Groups	Mean ± SD	Mean ± SD	Mean ± SD	Mean ± SD	Mean ± SD	
**Group A**	86.78 ± 15.37	47.74 ± 11.54	90.28 ± 11.64	89.04 ± 11.48	88.46 ± 9.59	*P* = 0.08
**Group B**	85.26 ± 12.99	86.96 ± 10.37	85.66 ± 6.61	81.81 ± 6.56	84.92 ± 7.96	*P* < 0.001
**Group C**	87.73 ± 13.36	86.96 ± 10.84	82.69 ± 10.04	79.90 ± 9.59	83.81 ± 8.85	*P* < 0.001
**Total**	85.91 ± 13.03	87.21 ± 10.85	86.18 ± 10.04	83.53 ± 10.11	85.73 ± 8.74	
**Effect Size**	0.11	3.61	0.75	0.90	0.53	
***P*-Value**^b^	*P* = 0.84	*P* = 0.91	*P* < 0.001¥	*P* < 0.001¥		

^a^The repeated measures ANOVA

^b^The one-way ANOVA

¥: According to Tukey’s post-hoc test: There was a significant difference between the mean total scores of sexual quality of life and Group A-C and Group A-B (*P* < 0.05) but there was no significant difference between the mean total scores of sexual quality of life and Group B-C (*P* > 0.05)

The present findings show that the number of pregnant women subject to sexual violence in group A decreased significantly from 30% (*n* = 15) before training to 14% (*n* = 7) at the end of pregnancy (after the intervention). The number of pregnant women subject to sexual violence also decreased in group B from 20.8% (*n* = 11) before training to 13.2% (*n* = 7) at the end of pregnancy (after the intervention). The reduction in violence was higher in group A than group B, but in group C, the number of pregnant women subject to sexual violence increased from 9.8% (*n* = 5) before training to 15.7% (*n* = 87) at the end of pregnancy (after the intervention). There was a significant difference between the three groups in the mean sexual violence score in the third trimester and after the intervention (the end of the third trimester). Also, according to Cochrane’s test, the mean score of sexual violence experienced by the pregnant women in group B differed significantly over time, from the first trimester to the second and third and then through to the end of the third trimester (post-intervention) (*P* < 0.001) (Table 4).

**Table 4:** The trend of the total sexual violence scores in the different groups (A = 50, B = 53, C = 51) during pregnancy

Time	1st trimester	2nd trimester	3rd trimester	End of pregnancy	***P***-Value^a^
Groups	N (%)	N (%)	N (%)	N (%)	
**Group A**					
Sexually violated	15 (30)	11 (22)	6 (12)	7 (14)	*P* = 0.019
Not sexually violated	35 (70)	39 (78)	44 (88)	43 (86)	
Total	50 (100)	50 (100)	50 (100)	50 (100)	
**Group B**					
Sexually violated	5 (9.8)	4 (7.8)	8 (15.7)	8 (15.7)	*P* = 0.04
Not sexually violated	46 (90.2)	47 (92.2)	43 (84.3)	43 (84.3)	
Total	51 (100)	51 (100)	51 (100)	51 (100)	
**Group C**					
Sexually violated	11 (20.8)	4 (7.5)	14 (7.5)	7 (13.2)	*P* = 0.29
Not sexually violated	42 (79.2)	49 (92.5)	49 (92.5)	46 (86.8)	
Total	53 (100)	53 (100)	53 (100)	53 (100)	
***P*-Value**^b^	*P* = 0.04	*P* = 0.04	*P* = 0.433	*P* = 0.935	

^a^Cochrane’s test

^b^Chi-square test

## Discussion

The present findings showed a significant difference in the mean total score of PSRI between the three groups over time.

The group training method was more effective on sexual responses during the different phases of the research compared to self-training and no intervention. Similar to the results of this study, Navidian et al. conducted a semi-experimental study on 100 pregnant women (*n* = 50 per group) using the PSRI questionnaire. The results showed that sexual activity and responses increased significantly in the intervention group after sex education [29]. The results of another study conducted by Heidari et al. on three groups, including an intervention group consisting of couples, an intervention group consisting only of pregnant women and also a control group, showed that the mean total score of sexual function after the intervention was significantly higher among the couples in the intervention group than the controls. Nonetheless, the mean score of sexual function was similar in both intervention groups of couples and pregnant women, and the face-to-face training of pregnant women, even in the absence of their husbands, as well as the training of couples, increased the sexual function of pregnant women [30, 31]. Nejati et al. [32] and Ismaili et al. [33] found a significant difference between the mean score of sexual function in both their control and case groups 4 weeks after the intervention.

Similar to the present findings, Afshar et al. also carried out a two-session educational intervention on 88 pregnant women at a gestational age of 8–10 years and found that, after training, the mean total score of sexual function was significantly higher in the intervention group than the control group, which indicates the effect of education on the sexual function of people undergoing counseling and education compared to controls [34]. The results of a study by Bahadoran et al. showed that training affected the sexual functioning of couples as both group training and individual face-to-face training. Nevertheless, no significant difference was observed after the intervention between the two groups with the two different training methods, which indicates that there was no difference between the two teaching methods in terms of their effect on sexual function. Nonetheless, education changed the overall score of sexual function in the research subjects [13]. The results of the cited studies are similar to the present findings, as they also showed the effect of training on the sexual response of pregnant women. It can be concluded that training improves the sexual response of pregnant women. The cited studies were mostly cross-sectional research on the effects of training in one or at most three sessions during one trimester of pregnancy, and had examined participants’ sexual function after 4 weeks. Meanwhile, in the present study, pregnant women were trained based on a valid study design and ongoing intervention during different months of pregnancy in addition to other routine pregnancy care.

Contrary to the present findings, Wannakosit et al. (2010) stated in one study that statistical tests did not show a significant difference in sexual function between the control and intervention groups [35]. The reason for this discrepancy could be the type of questionnaire used in this study, which was researcher-made, compared to the standard PSRI questionnaire. This disparity in results could also be attributed to the short time and educational content (20 min per session), through which it is highly unlikely that the sexual behavior of individuals changes.

The sexual quality of life is an important part of quality of life. The sexual quality of life includes a set of activities related to one’s sexual and emotional relationship with the spouse and sexual satisfaction [36, 37]. The results of the present study showed that the mean score of the overall sexual quality of life increased in the group training group with gestational age, and the maximum score was achieved in the third trimester of pregnancy. This score decreased slightly at the end of pregnancy (after the intervention); however, it was still higher than the scores before the intervention and in the second trimester. The decreased mean score of the sexual quality of life at the end of pregnancy may be due to the increased maternal weight and protuberant abdomen and also to the reduction in self-esteem and body image. Many stressors lead to distress, such as anxiety about childbirth, changes in the marital relationship, restrictions on sex and concerns about body image and physical health. The growing abdomen causes a gradual change in self-confidence and self-image [3]. Self-image, which is defined as the person’s perception of her body, is one of the factors that changes under the influence of physical and psychological changes during pregnancy. These changes are acceptable for some pregnant women but worrying for others [38, 39]. The results of the present study revealed that the score of sexual quality of life increased significantly more in the group training group than the other two groups with gestational age, and at the end of the pregnancy, the score of sexual quality of life was higher in the self-training group than the control group. These results imply the positive impact of education, especially group training, on the sexual quality of life in pregnant women.

In the study by Pourkhiz et al. on 84 primiparous women in 2015, the sexual quality of life increased in the training group compared to the control group [40]. AJ et al. also conducted a cross-sectional longitudinal study using the SQOL questionnaire in each trimester of pregnancy without any training. The mean total score of sexual quality of life and its domains (psychosexual feelings, sexual and relationship satisfaction, self-worthlessness, and sexual repression) decreased with gestational age from the first trimester to the third trimester of pregnancy [41]. The results of a study similar to the present one showed the positive effect of training on the sexual quality of life in pregnant women [29]. Sex education improves the sexual quality of life from the perspective of pregnant women and their husbands.

In the present study, the number of pregnant women subject to sexual violence decreased significantly in the intervention groups (group training and self-training). This reduction was higher in the group training than the self-training group, which means that training, especially group training, has effectively reduced the experience of sexual violence in pregnant women at different phases of the intervention. Since sexual violence is usually perpetrated by spouses, training should be given directly to the spouses to adequately decrease the rate of sexual violence during pregnancy.

Regarding the effect of sex education on sexual violence in pregnant women, a semi-experimental study was conducted on the effect of educating fathers (i.e. modifying their expectations about the birth of their child) on the severity of domestic violence. Seventy-five spouses of pregnant women received training on attachment skills through four 90-min sessions held once a week. The results showed that the mean score of sexual violence had a statistically significant correlation between the control and training groups. The rate of sexual violence was lower in the trained spouses after the intervention (late pregnancy) compared to before the intervention (early pregnancy). Nevertheless, sexual violence increased in the control group in late pregnancy compared to early pregnancy, indicating the positive effect of educating spouses on the rate of sexual violence experienced by pregnant women [42].

In another study in Iran, Taghizadeh et al. divided 141 pregnant women who had been subject to domestic violence into two groups, including an intervention and a control group, and then trained the intervention group on problem-solving skills in four sessions. Contrary to the results of the present study, their results showed that the mean score of sexual violence was not significantly different between the intervention and control groups, as the risk difference between sexual violence before and after the intervention was 4.8% in the control group and 4.9% in the intervention group [43], which may be due to differences in sampling and teaching methods.

In the present study, group training was administered to pregnant women using the context-dependent package. The teaching material included physiological changes during pregnancy, correct beliefs about sex and sexual intercourse during pregnancy, and the importance of sexual and marital intimacy at this period in life. The training content indirectly attempted to establish better marital relations between the pregnant women and their husbands, and as a result, reduced the rate of sexual violence experienced during this period in the group training group. The outcome of this training can be perceived quite clearly in the results of the present study concerning the training group compared to the control group.

Almost any education, even though delivered in a specific period of time, was effective on the enhancement of sexual life. The educational package in this study was delivered as a part of pregnancy routine care, during all trimesters. Considering cultural sensitivities in receiving sex educations, this makes the current package more available and continues if implemented.

The strengths of the present research included the existence of three different groups (two intervention groups with two different training methods and one control group) and also the continuous training given from the beginning to the end of pregnancy, which increases the promotion of maternal sexual health.

One of the limitations of this study was the absence of the pregnant women’s spouses (due to administrative restrictions) during the training sessions, which was partially offset by the provision of a summary booklet of the training sessions to the control group and also by the women passing the booklets on to their husbands, which indirectly enhanced the effect of the teaching material.

Another limitation of this study was the taboo nature of any discussions about sexual issues in Iran as a traditional and religious society. Culturally, many Iranian couples feel too ashamed or embarrassed about discussing their sexual problems. One of the possible obstacles in measuring the effectiveness of sexual education in this study was the unscientific recommendations of some gynecologists and midwives who ban their patients from having sex during pregnancy, especially in the third trimester.

## Conclusion

The results of the present study showed the effectiveness of the training program designed to improve the different dimensions of sexual health during pregnancy (responses and sexual activity, sexual quality of life and sexual violence) in the groups receiving training (especially group training) compared to the control group. The results showed the positive effect of education, especially group training vs. self-training, on the score of sexual activity and responses and the sexual quality of life in pregnant women. Also, the frequency of sexual violence experienced during pregnancy decreased significantly in the intervention groups (group training and self-training). As an individual learning approach, in-person learning can help pregnant women express their sensitive problems more easily and thus receive more accurate answers. According to the present findings, maintaining and promoting the sexual health of pregnant women by providing sexual health training to them during pregnancy along with other services provided by health care providers (especially midwives) can help improve the overall quality of health care services.

## Data Availability

The datasets used and/or analyses during the current study are available from the corresponding author on reasonable request.

## Abbreviations

ANOVA: Analysis of variance
ICC: The intraclass correlation coefficient
NICE: The National Institute for Health and Clinical Excellence
PSRI: Pregnancy sexual response inventory
SQOL-F: Sexual quality of life-female
SDG: Sustainable development goals
SHEP: The sexual health education package

## References

[1] Giovanni. Corona, Lior. Lowenstein, Natalio. Cruz, Fabrizio. Palumbo, Beatrice. Cuzin, Francesca. Tripodi, et al. The ESSM Manual of Sexual Medicine. edition n, editor. Amsterdam: The European Society for Sexual Medicine (ESSM); 2015.

[2] Read J (2004). Sexual problems associated with infertility, pregnancy, and ageing. BMJ.

[3] Johnson CE (2011). Sexual health during pregnancy and the postpartum. J Sex Med.

[4] DeJong J, Jawad R, Mortagy I, Shepard B (2005). The sexual and reproductive health of young people in the Arab countries and Iran. Reprod Health Matters.

[5] Sossah L (2014). Sexual behavior during pregnancy: a descriptive correlational study among pregnant women. Eur J Adv Res Biol Life Sci.

[6] Bayrami R, Sattarzadeh N, Ranjbar Koocheksarai F, Pezeshki MZ (2009). Eevaluation of sexual behaviors and some of its related factors in pregnant women, Tabriz, IRAN 2005. Urmia Med J.

[7] Kontoyannis M, Katsetos C, Panagopoulos P (2012). Sexual intercourse during pregnancy. Health Sci J.

[8] Corbacioglu Esmer A, Akca A, Akbayir O, Goksedef BP, Bakir VL (2013). Female sexual function and associated factors during pregnancy. J Obstet Gynaecol Res.

[9] Efe H, Bozkurt M, Sahin L, Mutlu MF, Api M, Çetin A. The effects of pregnancy on the sexual life of Turkish women. Proceedings in Obstetrics and Gynecology. 2014;4(1):1–11. doi:10.17077/2154-4751.1245

[10] Larsen U, Lawoyin TO (2002). Male sexual behaviour during Wife's pregnancy and postpartum abstinence period in Oyo state. Nigeria J Biosoc Sci.

[11] Onah HE, Iloabachie GC, Obi SN, Ezugwu FO, Eze JN (2002). Nigerian male sexual activity during pregnancy. Int J Gynaecol Obstet.

[12] Farnam F, Pakgohar M, Mir-mohammadali M (2011). Effect of pre-marriage counseling on marital satisfaction of Iranian newlywed couples: a randomized controlled trial. Sex Culture.

[13] Bahadoran P, MohammadiMahdiabadzade M, Nasiri H, GholamiDehaghi A (2015). The effect of face-to-face or group education during pregnancy on sexual function of couples in Isfahan. Iran J Nurs Midwifery Res.

[14] Khosravi F, Haseminasab L, Abdollahi M (2008). Study of the incidence and outcomes of domestic violence among pregnant women referring to childbirth unit of Sanandaj hospitals Urmia. Med J.

[15] Maasoumi R, Lamyian M, Montazeri A, Azin SA, Aguilar-Vafaie ME, Hajizadeh EJR (2013). The sexual quality of life-female (SQOL-F) questionnaire: translation and psychometric properties of the Iranian version. Reprod Health.

[16] Naldoni LM, Pazmino MA, Pezzan PA, Pereira SB, Duarte G, Ferreira CH (2011). Evaluation of sexual function in Brazilian pregnant women. J Sex Marital Ther.

[17] Karimi A, Dadgar S, Afiat M, Rahimi N (2013). The effect of sexual health education on couples’ sexual satisfaction. Iran J Obstetr Gynecol Infertility..

[18] Doss BD, Rhoades GK, Stanley SM, Markman HJ, Johnson CA (2009). Differential use of premarital education in first and second marriages. J Fam Psychol.

[19] Solimany A (2016). Delpisheh a, khademi N, jafari nia B, sayehmiri K. prevalence of violence against women in during pregnancy in IRAN: a systematic review and meta-analysis. J Nurs Midwifery Urmia Univ Med Sci.

[20] Khadivzadeh T, Erfanian F (2011). Comparison of domestic violence during pregnancy with the pre-pregnancy period and its relating factors. Iran J Obstetr Gynecol Infertility.

[21] Rudge CV, Calderon IM, Dias A, Lopes GP, Barbosa AP, Maesta I (2009). Design and validity of a questionnaire to assess sexuality in pregnant women. Reprod Health.

[22] Rudge CVC, IdMP C, APMd A, Piculo F, MVC R, AMP B (2018). Score establishment and Brazilian Portuguese version of the pregnancy sexual response inventory (PSRI). Rev Bras Ginecol Obstet.

[23] Taheri-Tanjani P, MA (2016). Psychometric properties of the Persian version of the activities of daily living scale and instrumental activities of daily living scale in elderly. Mazandaran Univ Med Sci.

[24] Plitcha SB, EK (2013). Munro's Statistical Methods for Health Care Research.

[25] Symonds T, Boolell M, Quirk F (2005). Development of a questionnaire on sexual quality of life in women. J Sex Marital Ther.

[26] Ahmadi M, Rahnavardi M, Kiyani M, Purhoseingholi A, Asadzadeh F (2015). Study of predisposing factors for domestic violence among women. J Health Care.

[27] Wunsch G (2007). Confounding and control. Demogr Res.

[28] Skelly AC, Dettori JR, Brodt ED (2012). Assessing bias: the importance of considering confounding. Evid Based Spine-Care J.

[29] Navidian A, Kykhaee A, Imani M, Taimoori B, Soltani P (2017). The Effect of Group Sexual Counseling on the Sexual Response of Pregnant Women. Int J Womens Health Reprod Sci.

[30] Leveno KJ, Spong CY, Dashe JS, Casey BM, Hoffman BL, Cunningham FG, et al. Williams obstetrics, 25th edition: McGraw-Hill Education; 2018. ISBN: 1259644324, 9781259644320. Available online: https://books.google.com/books?id=mhOdAQAACAAJ&dq=Williams+obstetrics,+25th+edition:+McGraw-Hill+Education;+2018&hl=en&sa=X&ved=2ahUKEwi5wdeCl43wAhXUuXEKHcV1Cu8Q6AEwAHoECAIQAg

[31] Heidari M, Aminshokravi F, Zayeri F, Azin SA (2018). Effect of Sexual Education on Sexual Function of Iranian Couples During Pregnancy: A Quasi Experimental Study. J Reprod Infertility.

[32] Nejati B, Kazemi F, Masoumi SZ, Parsa P, Karami M, Mortazavi A (2017). Efficacy of sexual consultation based on PLISSIT model (permission, limited information, specific suggestions, intensive therapy) on sexual function among pregnant women: a randomized controlled clinical trial. J Isfahan Med Sch.

[33] Mahnaz E, Nasim B, Sonia O (2020). Effect of a structured educational package on women's sexual function during pregnancy. Int J Gynecol Obstetr Gynecol.

[34] Afshar M (2012). Mohammad-Alizadeh-Charandabi S, Merghti-Khoei ES, Yavarikia P. The effect of sex education on the sexual function of women in the first half of pregnancy: a randomized controlled trial. J Caring Sci.

[35] Wannakosit S, Phupong V (2010). Sexual behavior in pregnancy: comparing between sexual education group and nonsexual education group. J Sex Med.

[36] Tairawhiti District Health (2008). Sexual Health Over Tairawhiti Strategy S.H.O.T.S.

[37] Tsai T-F, Yeh C-H, Hwang TIS (2011). Female sexual dysfunction: physiology, epidemiology, classification. Eval Treat Urol Sci.

[38] Women and sexual and reproductive health" Australian Women’s Health Network. Available online by: http://awhn.org.au/wpcontent/uploads/2015/03/94_AWHNWomenSexualReproductiveHealthPositionPaper2012.pdf2012. Accessed 22 May 2018

[39] Berek JS. Berek & Novak's Gynecology. 15th. Philadelphia: Lippincott Williams & Wilkins; 2012. ISBN 10: 818473610XISBN 13: 9788184736106

[40] Pourkhiz Z, Mohammad-Alizadeh-Charandabi S, Mirghafourvand M, Haj-Ebrahimi S, Ghaderi F. Effect of pelvic floor muscle training on female sexual function during pregnancy and postpartum: A randomized controlled trial. Iran Red Crescent Med J. 2017;19(10). 10.5812/ircmj.63218.

[41] Nezal AJ, Fatemi Samii Rad, Mehri Kalhor, kobra hasanpour, Mahmood Alipour, Ali Montazeri. Sexual quality of life in pregnant women: a cross sectional study. Payesh. 2018;17(4):421–429.

[42] Setodeh S, Ghodrati F, Akbarzadeh M (2019). The efficacy of father attachment education on the severity of domestic violence in primegravida women. J Caring Sci.

[43] Taghizadeh Z, Pourbakhtiar M, Ghasemzadeh S, Azimi K, Mehran A. The effect of training problem-solving skills for pregnant women experiencing intimate partner violence: a randomized control trial. Pan Afr Med J. 2018;30. 10.11604/pamj.2018.30.79.14872 PMID: 30344863; PMCID: PMC6191243. 10.11604/pamj.2018.30.79.14872 PMC6191243 30344863

